# Basic Performance of highly sensitive Chemiluminescent Enzyme Immunoassay for β‐Amyloid 1‐40 and β‐Amyloid 1‐42

**DOI:** 10.1002/alz.088502

**Published:** 2025-01-09

**Authors:** Takuya Nakazawa, Yuji Sakurai, Yoko Shima, Yuka Imai, Katsumi Aoyagi, Hisashi Nojima

**Affiliations:** ^1^ Fujirebio Inc., Hachioji‐shi, Tokyo Japan; ^2^ Fujirebio Inc., Tokyo Japan; ^3^ FUJIREBIO IINC., Hachioji, Tokyo Japan

## Abstract

**Background:**

Alzheimer's disease (AD) is the most common cause of a decline in cognitive ability. The pathogenesis of AD involves the deposition of β‐amyloid (Aβ) plaques and the formation of neurotoxic oligomers of Aβ protein. This leads to neurofibrillary tangles composed of phosphorylated Tau proteins, neuroinflammation, loss of nerve cells and synapses, and ultimately dementia. Recent research suggests that the Aβ1‐42/Aβ1‐40 ratio in plasma can predict amyloid deposition in the brain. However, the small difference between amyloid‐positive and ‐negative subjects results in a very low total allowable error. Therefore, the assay system with high reproducibility is required. Here, we improved Aβ1‐40 and Aβ1‐42 assays in Lumipulse and report the basic performance.

**Method:**

The Lumipulse system is a fully automated chemiluminescent enzyme immunoassay platform that processes samples in approximately 30 minutes. This assay uses two monoclonal antibodies to capture and detect Aβ1‐40 or Aβ1‐42. Baseline performance was analyzed in a limited set of characterized patient samples. The analytical study included a series of plasma samples (K2EDTA) at various concentration levels, and each analyte was tested in multiple replicates, sized to suit the various parameters tested.

**Result:**

As shown previously on other LUMIPULSE assays, variability (within‐ and between‐run) was found to be low. An acceptable Limit of Quantification or functional sensitivity was obtained based on the evaluation of low Aβ1‐40 and Aβ1‐42 concentrated serum samples. No cross reactivity toward other Aβ fragments was observed. In the frozen‐thawed samples, no effect was observed for several times.

**Conclusion:**

These studies showed promising performance with less variability, suggesting that these Aβ assays could be useful in screening appropriate patients for AD therapy.

